# Glioblastoma: epidemiology, molecular pathogenesis, diagnosis, management, and therapeutic resistance

**DOI:** 10.1186/s43556-026-00467-8

**Published:** 2026-05-08

**Authors:** Ji-Yong Sung, Kihwan Hwang

**Affiliations:** Department of Neurosurgery, Seoul National University Bundang Hospital, Seoul National University College of Medicine, Seongnam-Si, Republic of Korea

**Keywords:** Spatio-temporal tumor heterogeneity, Tumor microenvironment and immune suppression, Clonal evolution and immune escape, Therapeutic resistance in glioblastoma, Molecular classification and biomarkers, Adaptive immunotherapy strategies

## Abstract

Glioblastoma (GBM) remains the most common and lethal primary malignant brain tumor in adults, with a median survival of approximately 15 months despite maximal multimodal therapy. The 2021 WHO classification has improved diagnostic precision by incorporating key molecular features, including EGFR amplification, TERT promoter mutation, PTEN loss, and MGMT promoter methylation. However, current standard of care treatments such as surgical resection, radiotherapy, temozolomide, and tumor treating fields have reached a therapeutic plateau, highlighting the urgent need for new therapeutic strategies. Although immunotherapy has transformed the treatment of several solid tumors, its clinical benefit in GBM remains limited. This limitation reflects not only low tumor mutational burden or blood brain barrier constraints, but also the profound spatial and temporal heterogeneity of the tumor. Distinct tumor regions exhibit diverse immune states, while ongoing clonal evolution dynamically reshapes antigenicity, immune recognition, and therapeutic response. In this review, we provide a comprehensive overview of glioblastoma, including epidemiology, molecular pathogenesis, diagnostic approaches, tumor microenvironment, intratumoral heterogeneity, and current therapeutic strategies. We further synthesize recent advances in spatial and longitudinal profiling technologies to describe the dynamic tumor immune ecosystem. We discuss how spatial compartmentalization and evolutionary processes collectively drive immune escape and therapeutic resistance, and highlight emerging strategies including adaptive immunotherapy, precision targeted delivery, and multimodal monitoring to overcome these challenges.

## Introduction

Glioblastoma (GBM) is the most common and aggressive primary malignant brain tumor in adults, accounting for nearly half of all diffuse gliomas and carrying a median overall survival of approximately 15 months despite maximal therapy [1, 2]. Since the establishment of the Stupp protocol-combining surgical resection, radiotherapy, and temozolomide-treatment outcomes have improved only marginally [3]. The incorporation of tumor treating fields has modestly extended survival in selected patients, yet durable disease control remains rare [4]. Over the past decade, advances in molecular neuropathology, culminating in the 2021 World Health Organization (WHO) classification [5], have redefined GBM as a molecularly stratified disease characterized by IDH-wildtype status and hallmark alterations such as EGFR amplification, TERT promoter mutation, chromosome 7 gain/10 loss, and MGMT promoter methylation [6, 7]. These refinements have enhanced diagnostic precision but have not fundamentally altered the grim prognosis of this malignancy.

In parallel, immunotherapy has revolutionized the management of melanoma, lung cancer, and renal cell carcinoma, raising hopes that immune checkpoint inhibitors (ICIs) could similarly transform outcomes in GBM [8–12]. However, multiple large-scale clinical trials have failed to demonstrate significant survival benefits in unselected GBM populations. Traditional explanations-including low tumor mutational burden, limited neoantigen availability, and the restrictive blood–brain barrier-only partially account for this resistance [13]. Importantly, the limited efficacy of immunotherapy in GBM cannot be fully explained by conventional factors such as low tumor mutational burden or blood–brain barrier constraints alone; rather, it likely reflects a deeper level of spatio-temporal disorganization within the tumor–immune ecosystem. Increasingly, evidence from spatial transcriptomics, single-cell sequencing, and longitudinal genomic profiling indicates that GBM is defined by profound spatial and evolutionary heterogeneity [14, 15]. Immune activity varies dramatically across tumor regions, from hypoxic, myeloid-dominated cores to intermittently inflamed invasive margins, while clonal diversification and immune editing dynamically reshape antigenicity over time. These interacting dimensions generate a moving and compartmentalized immune landscape that challenges conventional, static therapeutic approaches [16, 17].

**Fig. 1:**
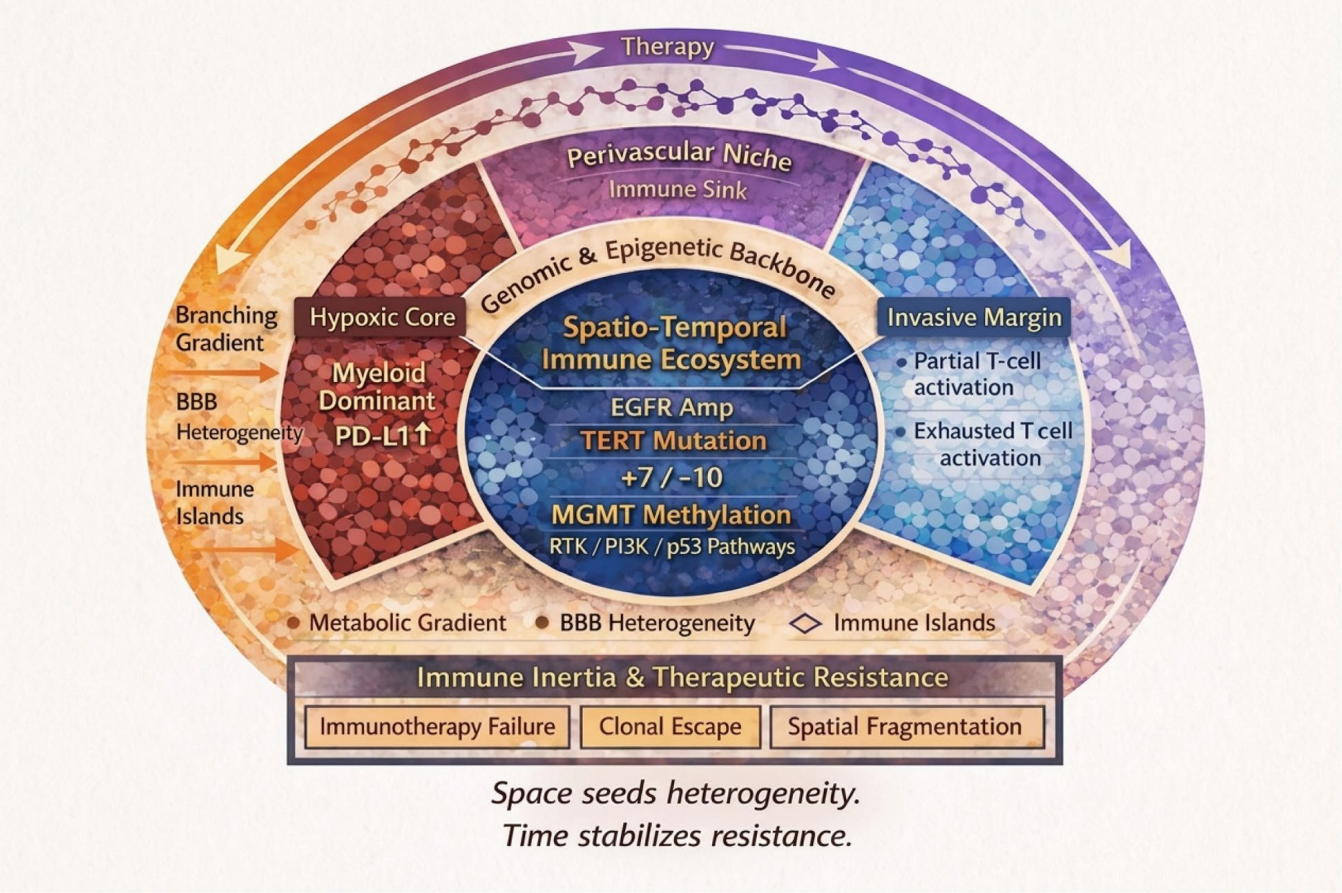
Glioblastoma as a spatio-temporal immune ecosystem. Glioblastoma (GBM) is conceptualized as a spatio-temporal immune ecosystem shaped by the interaction between a conserved molecular backbone, spatial immune compartmentalization, and dynamic clonal evolution. At the core lies the genomic and epigenetic backbone, defined by recurrent driver alterations including EGFR amplification, TERT promoter mutation, chromosome 7 gain/10 loss, MGMT promoter methylation, and dysregulation of RTK/PI3K, p53, and RB pathways. Although these trunk events are shared across tumor cells, intratumoral diversification generates distinct subclonal populations. Spatially, GBM exhibits structured immune heterogeneity. Hypoxic tumor cores are enriched for myeloid-dominant, PD-L1–expressing macrophages and profound metabolic stress, whereas invasive margins demonstrate partial T-cell activation accompanied by functional exhaustion. Perivascular niches function as immune sinks that modulate cellular trafficking and cytokine gradients. Blood–brain barrier heterogeneity, immune islands, and metabolic gradients further fragment immune accessibility across tumor regions. Temporally, branching clonal evolution and immune editing reshape antigenicity during and after therapy, promoting clonal escape and T-cell exhaustion. The interaction between spatial compartmentalization and evolutionary adaptation stabilizes immune inertia, ultimately resulting in therapeutic resistance. This integrated framework provides a conceptual basis for adaptive, ecosystem-informed neuroimmuno-oncology strategies

In this review, we provide a comprehensive overview of glioblastoma, encompassing epidemiology, molecular pathogenesis, diagnostic strategies, and current standards of care. Building upon this foundation, we synthesize emerging insights into spatial immune architecture and evolutionary clonal dynamics, proposing an integrated spatio-temporal immune ecosystem framework to explain therapeutic resistance. By linking molecular classification with immune topology and evolutionary adaptation, we aim to outline translational strategies for adaptive, precision-guided immunotherapy in GBM and to highlight future directions for overcoming its persistent clinical impasse (Fig. 1).

## Epidemiology and clinical landscape

Glioblastoma represents a clinically devastating malignancy with limited therapeutic progress despite decades of research. In this section, we outline the epidemiological patterns, survival outcomes, and population-level molecular features that define the clinical landscape of GBM and provide the foundation for understanding its biological complexity.

### Incidence, demographics, and global trends

Glioblastoma (GBM) represents the most common and malignant primary brain tumor in adults, accounting for approximately 45–50% of all diffuse gliomas and nearly 15% of all primary central nervous system (CNS) tumors [4]. Population-based registries, including the Central Brain Tumor Registry of the United States (CBTRUS) and European cancer surveillance networks [1], report an annual incidence of approximately 3–5 cases per 100,000 individuals [18]. Incidence increases markedly with age, peaking in the sixth to seventh decades of life, and is slightly higher in males than in females, with a male-to-female ratio of approximately 1.3–1.6:1 [19]. Geographic variation exists but remains modest compared with many systemic malignancies. Higher incidence rates are observed in North America and Northern Europe, while lower rates are reported in parts of Asia and Africa [20]. These differences may reflect variations in diagnostic access, imaging availability, genetic background, and environmental exposures rather than true biologic divergence. Known environmental risk factors are limited [21]; exposure to high-dose ionizing radiation remains the only well-established external risk factor. Most GBMs arise sporadically [22], although rare hereditary cancer syndromes-including Li-Fraumeni syndrome (TP53 mutations [23]), neurofibromatosis type 1 (NF1), and Lynch syndrome-are associated with increased glioma susceptibility [24]. Importantly, the majority of contemporary GBM cases correspond to IDH-wildtype diffuse astrocytic tumors under the 2021 WHO classification [5], which now integrates molecular features into the definition of GBM. This reclassification has refined epidemiologic reporting by distinguishing IDH-mutant astrocytomas from IDH-wildtype glioblastoma, thereby aligning epidemiology with molecular biology [25].

### Survival statistics and prognostic determinants

Despite advances in surgical technique, radiotherapy planning, and systemic therapy, glioblastoma remains associated with dismal survival outcomes. The median overall survival for newly diagnosed GBM is approximately 14–16 months with standard-of-care treatment [3], and the 5-year survival rate remains below 7%. In elderly patients (≥ 70 years), median survival is often less than 10 months, reflecting both biological aggressiveness and treatment limitations in this population [26]. Prognosis is influenced by a combination of clinical, radiographic, and molecular variables. Younger age, good performance status (e.g., Karnofsky Performance Status ≥ 70), maximal safe surgical resection, and completion of combined chemoradiotherapy are consistently associated with improved outcomes. Radiographic features such as extent of resection and absence of multifocal disease also correlate with survival [3]. Among molecular determinants, MGMT promoter methylation [7] is the most robust predictive biomarker, conferring increased sensitivity to temozolomide and improved survival. IDH mutation, although rare in classical GBM under current classification, historically defined a subset with markedly better prognosis and distinct biology. Additional alterations-including TERT promoter mutation, EGFR amplification, chromosome 7 gain/10 loss, and CDKN2A/B deletion-contribute to tumor behavior but exhibit variable prognostic value depending on context [27–29]. Notably, survival heterogeneity persists even among patients with similar molecular profiles, suggesting that additional layers of complexity-including intratumoral heterogeneity, immune landscape variability, and evolutionary dynamics-modulate clinical trajectories beyond static genomic markers.

### Molecular epidemiology and population-level genomic patterns

The integration of molecular profiling into routine diagnosis has reshaped the epidemiologic understanding of GBM. IDH-wildtype tumors constitute the overwhelming majority of cases and are characterized by early genetic events such as TERT promoter mutation, EGFR amplification, and chromosome 7 gain/10 loss [6]. These alterations are detectable in the majority of adult GBMs across populations, suggesting convergent evolutionary pathways in tumor initiation [30]. Transcriptomic subclassification has historically identified proneural, classical, and mesenchymal subtypes, each associated with distinct signaling networks and immune microenvironment features [31]. Although the clinical utility of these subtypes remains debated, population-level analyses reveal subtype-associated differences in immune infiltration, angiogenic activity, and response to therapy [32]. For example, mesenchymal-enriched tumors tend to display higher macrophage infiltration and inflammatory gene signatures, whereas proneural-like tumors often exhibit lower immune cell density but greater metabolic specialization [33]. At the population scale, large genomic consortia have demonstrated substantial intertumoral diversity, yet also recurrent pathway-level convergence involving RTK/PI3K signaling, p53 pathway disruption, and RB pathway inactivation. These recurrent alterations form the backbone of GBM molecular epidemiology; however, they coexist with extensive intratumoral and interregional heterogeneity [34]. This coexistence of population-level genomic consistency and tumor-level diversification foreshadows the central challenge explored in subsequent sections: while GBM shares common molecular hallmarks across patients, each tumor evolves as a spatially and temporally distinct ecosystem [35, 36].

## Molecular pathogenesis and classification

The biological behavior of glioblastoma is driven by a complex interplay of genetic, epigenetic, and metabolic alterations. This section summarizes the core molecular architecture of GBM, highlighting the key pathways and cellular states that underpin tumor initiation, progression, and therapeutic resistance.

### WHO 2021 classification and molecular definition

The understanding of GBM pathogenesis has been fundamentally reshaped by the integration of molecular diagnostics into neuropathological classification. The 2021 World Health Organization (WHO) classification of central nervous system tumors formally defines glioblastoma as an IDH-wildtype diffuse astrocytic tumor in adults that demonstrates one or more of the following molecular features [5, 37, 38]: TERT promoter mutation, EGFR amplification, or combined whole chromosome 7 gain and chromosome 10 loss (+7/–10), even in the absence of histologic necrosis or microvascular proliferation. This molecularly anchored definition reflects a paradigm shift-from morphology-based diagnosis to genotype-informed classification. This redefinition distinguishes IDH-mutant astrocytomas (formerly secondary GBMs) from IDH-wildtype glioblastomas, acknowledging their divergent biology, epidemiology, and clinical behavior. IDH-wildtype GBM represents a genetically aggressive entity characterized by early telomere maintenance activation, receptor tyrosine kinase (RTK) signaling amplification, and genomic instability. Thus, molecular classification not only improves diagnostic precision but also captures the fundamental biological architecture of GBM initiation.

### Core genetic drivers and signaling pathways

At the genomic level, GBM is defined by recurrent alterations converging on three principal pathways: RTK/PI3K signaling, the p53 axis, and the RB cell-cycle pathway [39]. The RTK/PI3K pathway is the most frequently dysregulated signaling network in GBM. EGFR amplification, mutation (including EGFRvIII), and overexpression occur in approximately 40–50% of cases, driving constitutive activation of downstream PI3K–AKT–mTOR signaling [35]. PTEN loss further enhances pathway activation, promoting proliferation, metabolic adaptation, and resistance to apoptosis. Additional RTKs, including PDGFRA and MET, contribute to intertumoral variability and subtype specification [40]. Disruption of the p53 pathway occurs through TP53 mutation, MDM2 amplification, or ARF deletion, impairing DNA damage response and apoptotic regulation. In parallel, RB pathway dysregulation-commonly via CDKN2A/B deletion or RB1 mutation-facilitates unchecked cell-cycle progression. Together, these core pathway alterations establish a proliferative, apoptosis-resistant cellular state that underlies rapid tumor expansion. TERT promoter mutations, present in the majority of IDH-wildtype GBMs, enable telomerase reactivation and replicative immortality [41]. Chromosomal instability, characterized by widespread copy number alterations and structural rearrangements, further fuels genetic diversity and subclonal diversification [42]. Importantly, while these alterations are recurrent across patients, their intratumoral distribution is heterogeneous. Distinct subclones within a single tumor may harbor different combinations of RTK amplifications or pathway activations, providing a substrate for spatially variable signaling outputs and therapy resistance.

### Epigenetic regulation and MGMT promoter methylation

Beyond genetic mutations, epigenetic dysregulation plays a central role in GBM pathogenesis. DNA methylation, histone modification, and chromatin remodeling collectively shape transcriptional programs that govern cell identity, differentiation state, and therapy responsiveness. The methylation status of the MGMT promoter is the most clinically relevant epigenetic biomarker in GBM. MGMT encodes O6-methylguanine-DNA methyltransferase, a DNA repair enzyme that reverses alkylating damage induced by temozolomide [7]. Promoter methylation silences MGMT expression, rendering tumor cells more susceptible to alkylating chemotherapy and conferring improved survival. However, MGMT methylation does not prevent tumor recurrence, reflecting the persistence of resistant subclones and adaptive evolution. Genome-wide methylation profiling has also identified distinct epigenetic landscapes associated with transcriptional subtypes and immune phenotypes [36]. Although the glioma-CpG island methylator phenotype (G-CIMP) is predominantly associated with IDH-mutant gliomas, epigenetic variability within IDH-wildtype GBM contributes to transcriptional plasticity and lineage state transitions [43]. Epigenetic reprogramming further influences immune visibility by regulating expression of antigen-presentation machinery, interferon-response genes, and cytokine networks. Thus, epigenetic plasticity provides an additional layer of adaptability that complements genetic evolution [44, 45].

### Metabolic reprogramming and hypoxia

Metabolic reprogramming is a defining hallmark of GBM pathogenesis. Rapid proliferation, aberrant vasculature, and insufficient perfusion generate regions of hypoxia and metabolic stress within the tumor microenvironment. Hypoxia-inducible factors (HIF-1α and HIF-2α) orchestrate adaptive transcriptional programs that enhance glycolysis, angiogenesis (via VEGF), and invasion. GBM cells exhibit enhanced aerobic glycolysis (the Warburg effect) [46], increased lipid metabolism, and altered mitochondrial function, enabling survival under fluctuating oxygen and nutrient availability. These metabolic adaptations are not uniformly distributed; rather, they exhibit spatial gradients, with hypoxic cores displaying distinct transcriptional and immune signatures compared with more perfused margins [47]. Metabolic stress also shapes the immune microenvironment [48]. Lactate accumulation, adenosine signaling, and altered arginine metabolism contribute to macrophage polarization and T-cell dysfunction. Therefore, metabolic reprogramming links tumor-intrinsic signaling to microenvironmental immune suppression, integrating oncogenic and immunologic pathogenesis [49, 50].

### Glioma stem-like cells and cellular plasticity

A critical component of GBM molecular pathogenesis is the presence of glioma stem-like cells (GSCs), a subpopulation endowed with self-renewal capacity, multilineage differentiation potential, and heightened resistance to therapy. GSCs reside preferentially within specialized niches-perivascular regions and hypoxic zones-where signaling interactions with endothelial cells, astrocytes, and immune cells sustain stemness programs [51, 52]. Key signaling pathways governing GSC maintenance include Notch, Wnt/β-catenin, Hedgehog, and STAT3 [53–56]. These pathways promote cellular plasticity and enable dynamic state transitions between stem-like and differentiated phenotypes [57]. Such plasticity underlies tumor recurrence following therapy, as treatment-resistant GSC populations repopulate the tumor mass [58]. Recent single-cell and spatial transcriptomic analyses have demonstrated that GBM comprises a continuum of cellular states rather than fixed subtypes [59]. Cells can transition between proneural-like, mesenchymal-like, and proliferative states in response to microenvironmental cues and therapy-induced stress [34]. This phenotypic fluidity is a central driver of evolutionary adaptation [50, 60, 61]. Collectively, the molecular pathogenesis of GBM is characterized by recurrent driver alterations, epigenetic plasticity, metabolic adaptation, and stem-like cellular hierarchies. While these mechanisms define tumor initiation and growth at the molecular level, they do not operate uniformly across the tumor mass or remain static over time. Instead, they generate a biologically diverse and dynamically evolving system-one in which genetic, epigenetic, and metabolic programs interact with microenvironmental forces to produce spatial and evolutionary heterogeneity [62, 63]. From this perspective, genetic alterations, epigenetic plasticity, metabolic reprogramming, and immune heterogeneity should not be viewed as independent processes, but as interdependent layers of a unified and evolving tumor ecosystem. Understanding this molecular foundation is essential before examining how these processes manifest as structured immune landscapes and clonal trajectories in subsequent sections.

## Diagnosis and biomarker-based monitoring

Accurate diagnosis and longitudinal monitoring of glioblastoma remain critical yet challenging due to its spatial and temporal heterogeneity. Here, we review current diagnostic modalities and emerging biomarker strategies, emphasizing their limitations and the need for integrated, multi-modal approaches.

### Radiographic and imaging advances

Neuroimaging remains the cornerstone of glioblastoma diagnosis and disease monitoring. Magnetic resonance imaging (MRI), including contrast-enhanced T1-weighted imaging, T2/FLAIR sequences, diffusion-weighted imaging, and perfusion imaging, provides essential information regarding tumor extent, necrosis, vascular permeability, and treatment response. However, conventional MRI primarily reflects structural alterations and fails to capture the underlying molecular and immune heterogeneity described in previous sections. Advanced imaging modalities-such as dynamic susceptibility contrast (DSC) perfusion MRI, diffusion tensor imaging (DTI), MR spectroscopy, and positron emission tomography (PET) using amino acid tracers-offer deeper insights into metabolic gradients, cellular density, and proliferative activity [64, 65]. Radiomics approaches, which extract high-dimensional quantitative features from imaging datasets, have begun to correlate radiographic texture patterns with molecular subtypes, MGMT methylation status, and immune infiltration signatures [66–69]. Despite these advances, imaging remains an indirect surrogate of tumor biology. Radiographic stability may coexist with ongoing clonal evolution, and apparent progression may reflect treatment-related inflammation (pseudoprogression) rather than true tumor growth. Thus, imaging alone is insufficient to fully characterize the dynamic spatio-temporal landscape of GBM [70, 71].

### Tissue-based molecular diagnostics

Histopathologic examination and molecular profiling of resected tumor tissue remain the gold standard for definitive diagnosis. The integration of IDH mutation testing, MGMT promoter methylation analysis [72], TERT promoter mutation assessment, and evaluation of EGFR amplification or chromosome 7 gain/10 loss has refined classification and prognostication under the WHO 2021 framework [5]. More recently, next-generation sequencing, single-cell RNA sequencing, and spatial transcriptomics have expanded the depth of tissue-based characterization. These approaches allow mapping of intratumoral heterogeneity, identification of glioma stem-like cell populations, and profiling of immune cell composition at unprecedented resolution [73]. However, tissue sampling presents inherent limitations. Surgical specimens represent only a fraction of the tumor mass and are often biased toward contrast-enhancing core regions. Infiltrative margins, which may harbor distinct immune or genetic states, are frequently under-sampled. Consequently, a single biopsy may underestimate both spatial diversity and resistant subclones [74].

### Limitations of serial biopsy

Longitudinal monitoring of clonal evolution ideally requires repeated tissue sampling. In practice, serial biopsy in GBM is constrained by significant clinical and ethical challenges [75]. Repeated neurosurgical procedures carry risks of neurological deficit, hemorrhage, infection, and functional decline. Moreover, many recurrent tumors arise in eloquent or deep brain regions that are not safely accessible. Even when repeat resection is performed at recurrence, sampling bias persists. Recurrent tumors may exhibit heterogeneous transformation across regions, and limited sampling may fail to capture the full spectrum of emerging subclones or immune alterations. Additionally, the blood–brain barrier restricts immune cell trafficking and therapeutic penetration in a spatially uneven manner, complicating interpretation of tissue-based immune profiling. Therefore, while tissue biopsy remains indispensable, it cannot provide continuous, global surveillance of tumor evolution [76]. These limitations underscore the need for minimally invasive strategies capable of capturing dynamic molecular changes across space and time. Despite the theoretical appeal of evolution-informed or adaptive therapeutic strategies, their clinical implementation in glioblastoma is constrained by practical sampling limitations. Unlike malignancies such as cutaneous melanoma, serial multi-site biopsies are rarely feasible in GBM due to surgical risk, eloquent brain regions, and patient morbidity. Consequently, continuous spatially resolved tissue sampling cannot serve as the primary monitoring framework. Moreover, while circulating tumor DNA analysis has emerged as a promising minimally invasive tool in systemic cancers, the blood–brain barrier significantly limits the sensitivity of plasma-based assays in GBM. Tumor-derived DNA fragments are often present at very low abundance in peripheral blood, reducing reliability for real-time clonal tracking. Therefore, adaptive therapeutic modulation in GBM must rely on integrated multimodal inference rather than repeated invasive sampling. Imaging-based biomarkers, cerebrospinal fluid (CSF)–derived ctDNA, immune profiling when available, and computational modeling of partial data collectively provide a more realistic framework for longitudinal monitoring [77]. These approaches aim to approximate clonal and immune dynamics without requiring frequent multi-regional biopsies [78].

### Liquid biopsy and circulating biomarkers

Liquid biopsy has emerged as a promising approach for longitudinal monitoring of GBM. Circulating tumor DNA, cell-free DNA, extracellular vesicles, circulating tumor cells, and T-cell receptor (TCR) sequencing represent potential tools for non-invasive assessment. In contrast to systemic malignancies, detection of ctDNA in plasma from GBM patients is often limited by the BBB and relatively low tumor DNA shedding into peripheral circulation [79]. However, cerebrospinal fluid (CSF)–derived ctDNA [80] demonstrates higher sensitivity and may better reflect intracranial tumor genetics. CSF-based liquid biopsy can detect mutations in TERT, EGFR, TP53, and other driver genes, and may reveal emerging resistance alterations prior to radiographic progression. Longitudinal ctDNA analysis enables tracking of clonal dynamics, mutational burden changes, and therapy-induced hypermutation [81]. Similarly, circulating TCR repertoire analysis can provide insights into immune diversity and exhaustion states over time. While standardization and sensitivity improvements are still needed, liquid biopsy represents a critical step toward real-time evolutionary monitoring in GBM [82].

### Integrated multi-modal monitoring strategies

Future diagnostic paradigms must integrate imaging, tissue profiling, and liquid biopsy into a unified monitoring framework. Spatial transcriptomics [83] and multiplex immune imaging [84] can map regional immune niches at baseline, while longitudinal ctDNA and TCR sequencing can track temporal clonal shifts. Computational integration of these multimodal datasets may allow construction of dynamic tumor atlases-models that predict which spatial regions harbor high-risk subclones or impending immune escape [85]. Machine learning approaches can correlate radiomic features with molecular evolution, potentially enabling non-invasive inference of spatial immune heterogeneity. By combining structural imaging, molecular diagnostics, and circulating biomarkers, clinicians may eventually move from static snapshot assessments to continuous surveillance of GBM as an evolving ecosystem [82]. Such integrative monitoring will be essential for implementing adaptive, evolution-informed therapeutic strategies discussed in subsequent sections.

### Technological and methodological limitations

Despite remarkable advances in single-cell sequencing, spatial transcriptomics [86], and multi-omic integration, several technological limitations constrain our ability to fully capture the spatio-temporal immune ecosystem of GBM. While these studies have significantly advanced our understanding of GBM biology, most remain limited by single time-point sampling and region-specific analyses, thereby potentially overlooking the continuous and system-level dynamics that govern tumor evolution.

First, paired spatial and longitudinal datasets remain scarce [86]. Most spatial transcriptomic studies rely on single time-point surgical specimens, limiting insight into how regional immune niches evolve during treatment and recurrence. Without temporally matched spatial sampling, it is difficult to disentangle true evolutionary shifts from baseline regional heterogeneity. Second, sampling-site heterogeneity introduces significant interpretative bias [87]. Surgical specimens are frequently obtained from contrast-enhancing core regions, whereas infiltrative margins and non-enhancing areas are underrepresented. Given the steep immune and metabolic gradients across tumor compartments, molecular and immune profiling derived from a single region may incompletely reflect the global tumor ecosystem. Third, the blood–brain barrier (BBB) imposes both biological and technical constraints [88]. The heterogeneous integrity of the BBB affects immune cell trafficking, therapeutic delivery, and the sensitivity of circulating biomarker detection, particularly plasma-derived ctDNA [79]. As a result, peripheral blood–based assays may underestimate clonal diversity and immune dynamics occurring within spatially shielded tumor regions [80]. Collectively, these limitations highlight that current technologies provide high-resolution snapshots rather than continuous, ecosystem-level surveillance. Future progress will depend on integrating spatial mapping, cerebrospinal fluid–based biomarkers, advanced imaging, and computational modeling to approximate dynamic tumor–immune interactions across both space and time. Notably, even high-resolution technologies such as single-cell sequencing and spatial transcriptomics provide only fragmented snapshots of a continuously evolving system, underscoring the need for integrative and longitudinal frameworks.

## Tumor microenvironment and immune landscape

Beyond tumor-intrinsic alterations, glioblastoma is shaped by a complex and highly immunosuppressive microenvironment. This section examines the cellular and molecular composition of the tumor microenvironment, focusing on immune regulation, vascular interactions, and spatially organized immune states.

### Cellular composition of the microenvironment

Glioblastoma is not composed solely of malignant astrocytic cells; rather, it represents a complex multicellular ecosystem in which tumor cells coexist with immune cells, endothelial cells, pericytes, astrocytes, and extracellular matrix components. Immune cells constitute up to 30–50% of the tumor mass, yet their composition and functional orientation differ markedly from those observed in immunotherapy-responsive cancers. The dominant immune population within GBM consists of tumor-associated macrophages (TAMs), which include both brain-resident microglia and bone marrow–derived macrophages (BMDMs) [89]. These cells display extensive transcriptional plasticity but are frequently skewed toward immunosuppressive, M2-like phenotypes characterized by expression of CD163, CD206, ARG1, IL-10, and TGF-β. In contrast, cytotoxic CD8⁺ T cells are relatively sparse and often localized to perivascular or marginal regions. Regulatory T cells (Tregs) and myeloid-derived suppressor cells (MDSCs) [90] further contribute to immune suppression [91, 92]. Endothelial cells and pericytes form aberrant, highly permeable vasculature, while reactive astrocytes and fibroblast-like stromal cells participate in cytokine signaling and matrix remodeling. Together, these components create a structurally and functionally integrated tumor microenvironment in which malignant and non-malignant cells cooperate to sustain growth and immune evasion.

### Immune checkpoint networks and T-cell dysfunction

Although immune checkpoint inhibitors have transformed cancer therapy in peripheral malignancies, GBM exhibits limited responsiveness. This resistance reflects not the absence of immune signaling, but rather its dysregulated and compartmentalized nature. GBM cells and infiltrating myeloid populations frequently express PD-L1, particularly under hypoxic conditions mediated by HIF-1α signaling [93]. In parallel, tumor-infiltrating lymphocytes display high levels of inhibitory receptors including PD-1, TIM-3, LAG-3, and TIGIT. Chronic antigen exposure within an immunosuppressive cytokine milieu drives T-cell exhaustion, characterized by diminished effector cytokine production, impaired proliferation, and sustained expression of transcription factors such as TOX and NR4A [94]. In addition to checkpoint receptor–ligand interactions, GBM suppresses immune activation through defective antigen presentation. Downregulation of MHC class I molecules, loss of β2-microglobulin, and impaired interferon signaling reduce tumor immunogenicity. Consequently, immune checkpoint blockade alone often fails to restore functional cytotoxic immunity, as exhausted T cells remain epigenetically fixed in dysfunctional states and tumor cells remain partially invisible.

### Myeloid dominance and immunosuppressive circuits

A defining feature of the GBM immune landscape is myeloid predominance. TAMs can constitute the majority of immune cells within the tumor and play central roles in establishing immunosuppressive circuits. These macrophages secrete anti-inflammatory cytokines (IL-10, TGF-β), express immune checkpoint ligands (PD-L1, IDO1), and metabolically deplete essential nutrients such as arginine through arginase activity. They also produce VEGF and matrix metalloproteinases, promoting angiogenesis and invasion [24]. Crosstalk between TAMs and glioma stem-like cells reinforces stemness programs and therapeutic resistance. Importantly, microglia and BMDMs are not functionally identical. Spatial transcriptomic studies demonstrate lineage-specific compartmentalization: microglia are enriched at tumor margins, whereas BMDMs accumulate in hypoxic cores and perivascular niches [95]. These distinct origins correspond to differential transcriptional states and immune-modulatory capacities. The net effect is the establishment of a myeloid-dominated environment in which cytotoxic T-cell activity is both numerically and functionally constrained. This myeloid bias is a major obstacle to effective immunotherapy and represents a critical therapeutic target.

### Blood–brain barrier and the vascular–immune interface

The blood–brain barrier (BBB) and blood–tumor barrier (BTB) impose additional layers of immune regulation unique to CNS tumors. Unlike peripheral tumors, GBM develops within a neurovascular unit characterized by tight endothelial junctions, astrocytic endfeet, and pericyte coverage [88]. Although the BBB becomes partially disrupted in regions of contrast enhancement, permeability is heterogeneous. Areas of leakage may permit limited immune cell and antibody entry but are frequently accompanied by profound local immunosuppression [96]. Conversely, infiltrative tumor margins often retain an intact BBB, limiting both immune surveillance and drug delivery [97]. Perivascular niches serve as critical hubs of immune regulation. Macrophage-rich cuffs around abnormal vessels can act as immune sinks, sequestering and suppressing T cells before they penetrate deeper tumor parenchyma. Vascular remodeling driven by VEGF and ANGPT2 signaling further generates oxygen and nutrient gradients that influence macrophage polarization and T-cell functionality [98–101]. Thus, the vascular–immune interface functions not merely as a physical barrier but as an active regulator of immune accessibility and distribution within GBM [102].

### Functional immune gradients

**Fig. 2:**
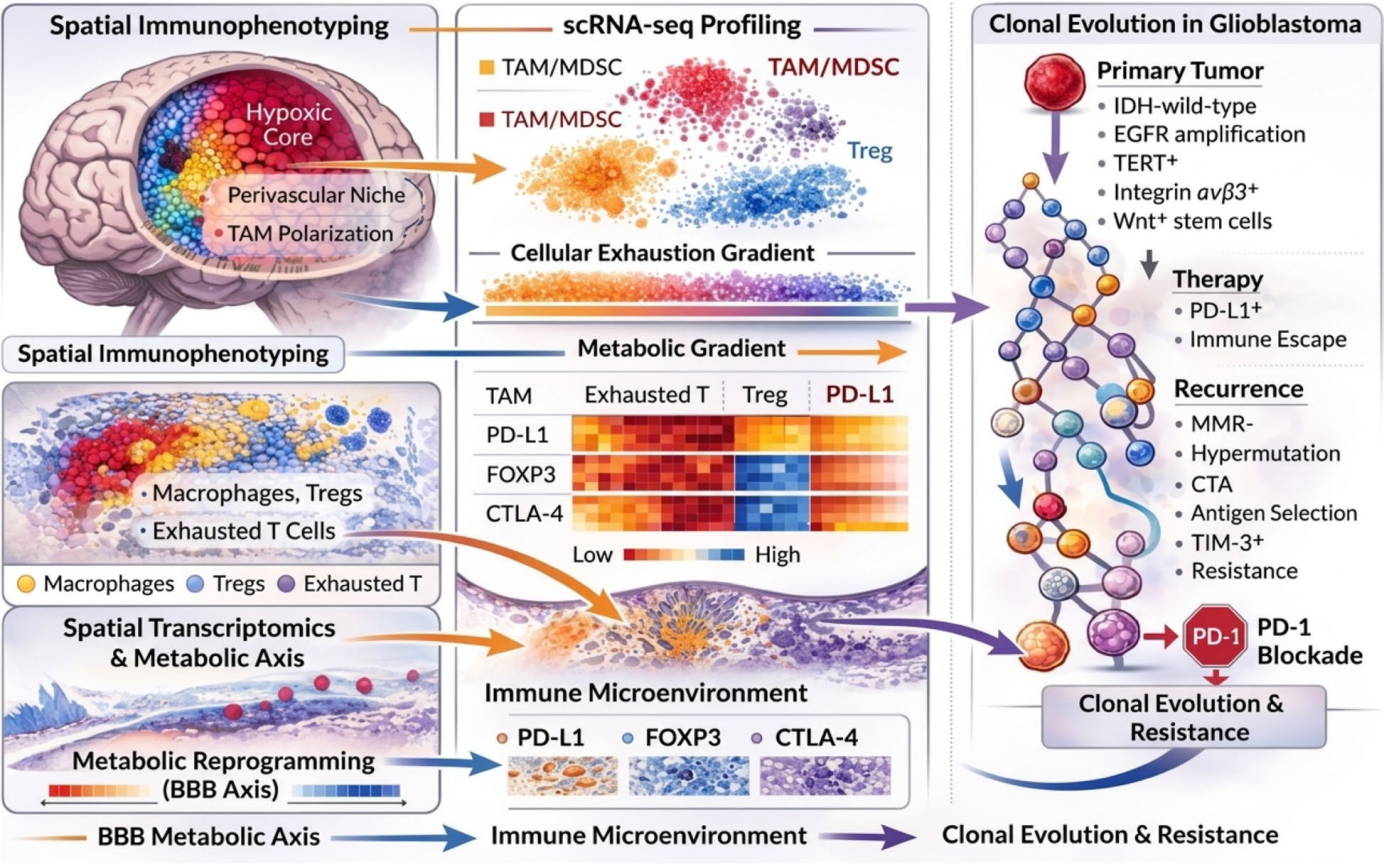
Spatial and evolutionary immune heterogeneity in glioblastoma. Glioblastoma (GBM) exhibits integrated spatial and evolutionary immune heterogeneity that shapes therapeutic resistance. Left panels illustrate spatial immunophenotyping of the tumor microenvironment. Hypoxic cores are enriched for tumor-associated macrophages (TAMs) and myeloid-derived suppressor cells (MDSCs), accompanied by PD-L1 upregulation and metabolic stress. Perivascular niches promote TAM polarization and function as immune-modulatory hubs. In contrast, invasive margins demonstrate partial T-cell activation, yet are characterized by progressive functional exhaustion. Middle panels depict single-cell RNA sequencing (scRNA-seq) and spatial transcriptomic analyses revealing discrete immune clusters, including TAM/MDSC populations, regulatory T cells (Tregs), and exhausted T cells. A cellular exhaustion gradient aligns with a metabolic gradient and blood–brain barrier (BBB) heterogeneity, forming a structured “BBB–metabolic axis” that fragments immune accessibility across tumor regions. Right panels represent branching clonal evolution during therapy. While primary tumors share trunk alterations (e.g., IDH-wildtype status, EGFR amplification, TERT activation), therapeutic pressure selects for PD-L1–high, immune-evasive subclones. Recurrent tumors demonstrate mismatch repair deficiency, hypermutation, antigenic selection, TIM-3 upregulation, and resistance to PD-1 blockade. Together, spatial compartmentalization and temporal clonal diversification converge to generate immune fragmentation and immunotherapy failure, underscoring the need for adaptive, ecosystem-informed treatment strategies

The GBM microenvironment is not immunologically uniform. Rather, it is structured by gradients of hypoxia, metabolic stress, cytokine concentration, and vascular density [103]. Hypoxic cores favor M2-like macrophage polarization and PD-L1 upregulation, while invasive edges may display partial interferon activation and intermittent T-cell engagement [104]. Single-cell and spatial transcriptomic analyses reveal that immune activation and suppression coexist within millimeters of tissue, forming a patchwork of immune niches [105]. Some regions exhibit interferon-stimulated gene expression and cytotoxic signatures, whereas others are deeply immune-cold. These spatially organized immune states are further reshaped over time by clonal evolution and therapy-induced stress. Consequently, the GBM tumor microenvironment must be understood not as a static immunosuppressive field, but as a dynamic and compartmentalized ecosystem [106, 107]. This recognition sets the stage for the next sections, which examine how spatial organization and evolutionary dynamics intersect to generate a spatio-temporal landscape of immune resistance. Collectively, these findings suggest that glioblastoma should not be interpreted as a uniformly immunosuppressive tumor, but rather as a dynamically organized system in which spatially distinct immune states coexist and interact, ultimately shaping therapeutic responsiveness (Fig. 2).

## Intratumoral heterogeneity and evolution

A defining feature of glioblastoma is its profound intratumoral heterogeneity and continuous evolutionary dynamics. In this section, we explore how spatial organization and microenvironmental pressures drive clonal diversification, immune escape, and therapeutic resistance.

### Spatial organization defines immune and metabolic niches

Glioblastoma exhibits pronounced spatial heterogeneity that extends beyond genetic diversity to encompass immune architecture, metabolic state, and vascular organization [108]. Histologically, GBM is structured into a hypoxic, necrotic core, a proliferative rim, and an infiltrative margin, each representing distinct biological microenvironments. The tumor core is typically characterized by severe hypoxia, metabolic stress, and enrichment of immunosuppressive tumor-associated macrophages, with limited cytotoxic T-cell infiltration and reduced antigen presentation capacity [15]. In contrast, the invasive margin retains relatively preserved vascular integrity and exhibits partial immune activation, with detectable CD8⁺ T cells and natural killer cells, although these populations frequently display features of functional exhaustion [109, 110].

Rather than forming uniform or concentric immune patterns, these regions establish spatially heterogeneous and functionally distinct immune niches. Recent spatial transcriptomic and imaging studies further reveal that immune activity is organized in a mosaic-like manner, with localized immune-active microdomains embedded within broader immunosuppressive territories. Within these niches, interactions among tumor cells, myeloid populations, and lymphocytes generate region-specific cytokine signaling, metabolic competition, and checkpoint regulation [111].

Collectively, this spatial organization gives rise to steep immune and metabolic gradients across the tumor, which in turn influence immune cell trafficking, therapeutic accessibility, and local treatment response. These structured microenvironments provide the ecological context in which distinct selective pressures emerge, setting the stage for subsequent clonal evolution and the development of therapeutic resistance. These spatially defined niches do not merely coexist but actively impose selective pressures that shape the evolutionary trajectories of tumor subclones.

### These niches impose selective pressures driving clonal evolution

The spatially defined immune and metabolic niches within glioblastoma create distinct selective pressures that shape tumor evolution. Variations in hypoxia, immune activity, and nutrient availability favor the expansion of subclones with region-specific adaptive advantages. As a result, GBM evolves through a branching pattern in which genetically and phenotypically diverse subclones emerge and occupy different spatial territories [76, 92].

Importantly, these evolutionary dynamics are closely linked to immune selection. Subclones capable of evading immune recognition—through reduced antigen presentation, altered interferon signaling, or other immune escape mechanisms—are preferentially maintained and expanded. Over time, this process leads to the progressive enrichment of less immunogenic and more therapy-resistant tumor populations. Thus, clonal evolution in GBM is not a uniform temporal process but a spatially structured phenomenon driven by heterogeneous microenvironmental pressures, ultimately contributing to immune escape and therapeutic resistance [112].

### Evolutionary dynamics lead to immune escape and therapeutic resistance

As clonal evolution progresses within spatially heterogeneous niches, tumor populations increasingly acquire mechanisms that enable immune evasion and treatment resistance. Immune editing plays a central role in this process, selectively eliminating highly immunogenic subclones while allowing the persistence and expansion of variants with reduced antigen presentation or impaired interferon signaling. Consequently, recurrent or treatment exposed tumors are often characterized by diminished immunogenicity and an increased capacity to evade immune surveillance [113].

In parallel, therapeutic interventions including radiotherapy, chemotherapy, and immunotherapy further intensify selective pressures, accelerating the emergence of resistant subclones. These adaptations may involve metabolic reprogramming, enhanced DNA repair, or reinforcement of immunosuppressive signaling pathways. Importantly, such resistance mechanisms are not uniformly distributed but remain shaped by the underlying spatial and evolutionary structure of the tumor [7].

Together, these processes establish a dynamic feedback loop in which spatial heterogeneity drives evolution, and evolution in turn reinforces immune escape and therapeutic resistance. This interconnected framework underscores the need to consider glioblastoma as a continuously evolving tumor immune ecosystem when designing effective treatment strategies.

## Management of glioblastoma

Despite advances in surgical and adjuvant therapies, glioblastoma remains largely incurable. This section reviews current standard treatments and highlights their inherent limitations, particularly in the context of tumor heterogeneity and adaptive resistance.

### Current standard of care and its limitations

#### Surgical resection

Maximal safe surgical resection remains the cornerstone of initial glioblastoma management. The primary goals of surgery are to reduce tumor burden, alleviate mass effect, obtain tissue for molecular diagnosis, and improve survival [114]. Numerous studies demonstrate a correlation between extent of resection and overall survival, particularly when gross total resection of contrast-enhancing tumor is achieved. However, surgical resection is inherently limited by the infiltrative nature of GBM [115]. Tumor cells extend microscopically beyond radiographic margins, infiltrating eloquent brain regions where aggressive resection risks irreversible neurological deficit. As a result, surgery primarily removes the proliferative core while leaving behind infiltrative subclones at the margin-regions that often harbor distinct genetic and immune states [116]. Thus, surgery reduces tumor bulk but does not eliminate spatial heterogeneity. Residual tumor cells serve as seeds for recurrence, frequently representing subpopulations with increased stemness, therapy resistance, or immune-evasive traits [117].

#### Radiotherapy

Postoperative radiotherapy, typically delivered as fractionated external-beam radiation to a total dose of 60 Gy, constitutes a critical component of the standard Stupp protocol. Radiation induces DNA damage, promotes tumor cell apoptosis, and may transiently enhance immunogenicity through increased antigen presentation and release of damage-associated molecular patterns (DAMPs) [3, 118]. Despite these effects, radiotherapy does not eradicate infiltrative tumor cells and may paradoxically contribute to evolutionary selection. Subclones with enhanced DNA repair capacity or altered cell-cycle regulation preferentially survive irradiation. Radiation-induced hypoxia and vascular remodeling can further modify the tumor microenvironment, potentially reinforcing immunosuppressive circuits [119, 120]. While radiotherapy may transiently modulate immune activity, its long-term impact often includes selection of resistant clones and alteration of immune niches rather than durable tumor control [121, 122].

#### Temozolomide chemotherapy

Temozolomide, an oral alkylating agent, is administered concurrently with radiotherapy and as adjuvant therapy [3]. Its cytotoxic effect depends on the induction of O6-methylguanine DNA lesions, which trigger mismatch repair–mediated apoptosis in tumor cells lacking functional MGMT repair activity [123]. MGMT promoter methylation remains the most robust predictive biomarker for TMZ responsiveness. Patients with methylated MGMT derive significant survival benefit, whereas those with unmethylated MGMT experience limited therapeutic gain [7]. However, even in responsive tumors, resistance inevitably develops. TMZ can induce a hypermutator phenotype, particularly in mismatch repair–deficient contexts. Rather than uniformly increasing immunogenicity, this process often accelerates clonal diversification and immune escape, as discussed in Sect. " Intratumoral heterogeneity and evolution". The selective pressure imposed by chemotherapy may favor subclones with altered DNA repair pathways, metabolic adaptations, or immune-evasive mechanisms. Therefore, TMZ contributes to both initial tumor control and subsequent evolutionary reshaping of the disease [124–126].

#### Tumor Treating Fields (TTF)

Tumor treating fields deliver alternating electric fields to disrupt mitotic spindle formation and inhibit tumor cell division. Clinical trials have demonstrated modest survival improvement when TTF is added to maintenance temozolomide therapy [2]. TTF is non-invasive and generally well tolerated; however, its mechanism primarily targets proliferating tumor cells and does not directly address immune suppression, clonal evolution, or spatial heterogeneity [127]. Its efficacy may therefore be constrained by non-dividing stem-like cells or deeply infiltrative subclones that evade field distribution [128]. While TTF extends survival in selected patients, it does not fundamentally alter the trajectory of recurrence [129].

### Immunotherapy and emerging therapeutic approaches

#### Immune Checkpoint inhibitors

Immune checkpoint inhibitors targeting PD-1, PD-L1, and CTLA-4 have transformed the management of multiple solid tumors; however, their efficacy in glioblastoma has been limited. Large randomized trials evaluating nivolumab in newly diagnosed and recurrent GBM (e.g., CheckMate 143, 498, and 548) failed to demonstrate a significant overall survival benefit compared with standard therapy [10–12]. Several factors contribute to this resistance. First, GBM typically exhibits a relatively low tumor mutational burden compared with melanoma or smoking-associated lung cancer, limiting neoantigen availability. Second, the tumor microenvironment is dominated by immunosuppressive myeloid populations and exhausted T cells [130]. Third, spatial compartmentalization restricts immune cell access to certain tumor regions. Finally, progressive immune editing and loss of antigen presentation further reduce tumor visibility. Notably, subsets of patients-such as those with mismatch repair deficiency or hypermutated recurrences-have shown occasional responses, suggesting that immunotherapy failure is not absolute but context-dependent [131]. These observations underscore the need for biomarker-guided patient selection and combination strategies [132]. Importantly, the interaction between standard-of-care therapies and immune checkpoint inhibitors (ICIs) has emerged as a critical determinant of therapeutic efficacy. Radiotherapy and certain chemotherapeutic agents are increasingly recognized not only for their cytotoxic effects but also for their immunomodulatory properties [133–135]. Radiotherapy can enhance anti-tumor immunity by promoting immunogenic cell death, increasing neoantigen release, and upregulating antigen presentation machinery, thereby functioning as an in situ vaccination strategy [12]. Local irradiation may also induce interferon signaling and augment T-cell priming within regional lymphoid structures. However, these immune-stimulatory effects are highly context-dependent and may be counterbalanced by radiation-induced lymphopenia or myeloid recruitment [136]. Similarly, selected chemotherapeutic agents have been shown to sensitize tumors to ICIs by increasing tumor immunogenicity, modulating myeloid polarization, or disrupting DNA damage response pathways that influence antigen presentation [137]. Recent evidence suggests that chemotherapy-induced genomic stress can transiently enhance immune visibility, thereby creating a therapeutic window for checkpoint blockade [138]. These findings indicate that ICIs should not be considered in isolation but rather as components of rational combination strategies in which radiotherapy and chemotherapy may reshape the immune landscape in ways that either facilitate or hinder immune reactivation. Optimizing the timing, sequencing, and spatial targeting of these modalities will be essential to translate immunologic synergy into durable clinical benefit in GBM.

#### Vaccine-based approaches

Therapeutic vaccines aim to stimulate tumor-specific immune responses by presenting tumor-associated antigens to the adaptive immune system [139]. Several strategies have been explored in GBM, including peptide vaccines targeting EGFRvIII [140], dendritic cell (DC) vaccines [141] loaded with tumor lysate, and personalized neoantigen vaccines. The EGFRvIII-targeted vaccine rindopepimut initially showed promise in early-phase trials but failed to improve survival in a phase III study, partly due to heterogeneous EGFRvIII expression and antigen loss at recurrence [142]. This highlighted a fundamental challenge: single-antigen targeting is vulnerable to evolutionary escape. Dendritic cell vaccines, such as autologous tumor lysate–loaded DC platforms, have demonstrated encouraging immunogenicity and modest survival signals in selected cohorts [143]. Personalized neoantigen vaccines [144], informed by tumor sequencing, represent a more adaptable approach but remain limited by tumor heterogeneity and dynamic antigen loss. Collectively, vaccine strategies demonstrate proof-of-concept immune activation but face significant barriers related to spatial antigen distribution and temporal clonal evolution.

#### Cellular therapies: CAR-T and TCR approaches

Adoptive cellular therapies, including chimeric antigen receptor T cells and T-cell receptor–engineered lymphocytes, offer targeted immune effector activity independent of endogenous antigen presentation. CAR-T therapies [145] targeting IL13Rα2, EGFRvIII, HER2 [146], and other GBM-associated antigens have shown evidence of tumor regression in individual cases. However, responses are often transient. Antigen heterogeneity, antigen loss, limited T-cell persistence, and immunosuppressive microenvironmental signals contribute to relapse [147]. In contrast to hematologic malignancies, GBM presents unique challenges: heterogeneous antigen expression across spatial regions, restricted trafficking across the blood–brain barrier, and rapid T-cell exhaustion within the CNS microenvironment. Strategies under investigation include multi-target CAR constructs, armored CAR-T cells engineered to secrete cytokines, and regional (intratumoral or intraventricular) delivery to overcome trafficking barriers. While promising, these approaches must contend with dynamic antigenic evolution and immune suppression [148, 149].

#### Oncolytic viruses and innate immune activation

Oncolytic viruses aim to selectively infect and lyse tumor cells while stimulating innate and adaptive immune responses [150]. Viral replication can induce immunogenic cell death, release tumor antigens, and activate pattern recognition receptor pathways such as cGAS–STING [151]. Clinical trials of oncolytic herpes simplex virus, adenovirus, and other viral platforms in GBM have demonstrated safety and localized immune activation. Some studies report increased T-cell infiltration and interferon signaling within treated regions. However, spatial delivery remains a challenge, and antiviral immunity may limit sustained viral replication. Moreover, immune activation may be restricted to injected areas, leaving distant tumor regions unaffected. Combination strategies integrating OVs with ICIs or targeted agents aim to amplify immune priming and overcome local immunosuppression [152, 153].

#### Lessons from clinical trials and mechanisms of resistance

The collective experience of immunotherapy trials in GBM reveals several recurring themes:

- Antigen Heterogeneity: Target antigens are variably expressed across spatial regions and may be lost during recurrence.
- Myeloid Dominance: Immunosuppressive macrophages and microglia attenuate T-cell activity.
- T-cell Exhaustion: Chronic antigen exposure drives dysfunctional immune states resistant to checkpoint blockade alone.
- Spatial Compartmentalization: Immune activation may occur in isolated niches without systemic tumor control.
- Evolutionary Adaptation: Tumors dynamically reshape their antigenic and signaling landscapes under therapeutic pressure.

Importantly, immunotherapy failure in GBM does not imply immunologic irrelevance. Instead, it reflects the complexity of a spatially structured and evolutionarily adaptive tumor ecosystem [154]. Successful immunotherapy will likely require:

- Multi-antigen targeting to mitigate antigen loss
- Myeloid reprogramming strategies (e.g., CSF1R, PI3Kγ, STAT3 inhibition)
- Spatially guided delivery (e.g., convection-enhanced delivery, focused ultrasound)
- Evolution-informed adaptive scheduling

In this context, immunotherapy should not be viewed as a standalone intervention but as a component of a dynamic, integrated treatment strategy responsive to spatial and temporal tumor dynamics [155–158]. The limited success of immunotherapy in GBM reflects the convergence of spatial immune fragmentation and evolutionary clonal adaptation. Neither axis alone fully explains resistance; rather, their interaction produces a continuously shifting immune-resistance landscape. In the following section, we integrate these dimensions into a unified spatio-temporal immune ecosystem framework, proposing a conceptual model to guide adaptive, precision neuroimmuno-oncology.

### Why standard therapy fails: a spatio-temporal perspective

Despite multimodal treatment, nearly all GBMs recur, typically within 6–9 months of completing therapy. Recurrence is rarely a simple regrowth of the original dominant clone [159, 160]. Instead, it reflects the expansion of residual subclonal populations that have survived surgical debulking, radiation-induced DNA damage, and chemotherapy-induced stress [161]. From a spatial perspective, residual tumor cells at infiltrative margins or within hypoxic niches are relatively protected from maximal resection and may experience sublethal therapy exposure [162, 163]. These regions can act as sanctuaries for resistant subclones. From a temporal perspective, therapy itself imposes selective pressure, reshaping the clonal architecture of the tumor. Hypermutation, epigenetic reprogramming, metabolic adaptation, and immune editing collectively drive the emergence of recurrent tumors that are genetically and immunologically distinct from their primaries [164]. Thus, failure of standard therapy is not merely due to insufficient cytotoxic intensity, but rather to the persistence of spatially shielded and evolutionarily adaptable tumor ecosystems. Conventional treatments target proliferative bulk disease but do not fully address immune suppression, cellular plasticity, or dynamic clonal diversification. Recognizing these limitations provides the rationale for integrating immunotherapy, spatially targeted delivery systems, and evolution-informed adaptive strategies in the next generation of GBM management [165].

### Mechanisms of therapeutic resistance

Therapeutic resistance in glioblastoma arises from a convergence of tumor-intrinsic and microenvironmental mechanisms that collectively impair effective anti-tumor immunity. While the spatio-temporal framework described above explains treatment failure at a systems level, these failures are ultimately mediated by specific biological processes operating at cellular and molecular scales. One major mechanism is immune evasion through impaired antigen presentation. Glioblastoma cells frequently downregulate major histocompatibility complex (MHC) class I molecules and exhibit defects in interferon signaling pathways, thereby reducing tumor immunogenicity and limiting T-cell recognition. In parallel, chronic antigen exposure within an immunosuppressive microenvironment drives T-cell exhaustion. Tumor-infiltrating lymphocytes display sustained expression of inhibitory receptors such as PD-1, TIM-3, and LAG-3, accompanied by diminished effector function and proliferative capacity. These dysfunctional states are often epigenetically stabilized, limiting reinvigoration by immune checkpoint blockade. Myeloid-driven immunosuppression represents another dominant axis of resistance. Tumor-associated macrophages and other myeloid populations suppress cytotoxic T-cell activity through secretion of immunosuppressive cytokines, including IL-10 and TGF-β, as well as through metabolic competition and checkpoint ligand expression. These cells form a regulatory network that reinforces immune suppression across multiple tumor regions. Finally, clonal evolution under therapeutic and immune pressure promotes the emergence of resistant subpopulations. Immune editing and antigen loss reduce the effectiveness of targeted and immune-based therapies, while ongoing genetic and epigenetic diversification enables adaptive escape. Importantly, these mechanisms do not operate in isolation but are dynamically shaped by spatial heterogeneity and temporal evolution, collectively sustaining resistance across the glioblastoma ecosystem.

### Translational and clinical challenges

#### Blood–brain barrier constraints

The blood–brain barrier remains one of the most formidable obstacles in glioblastoma therapy. Although contrast-enhancing regions demonstrate partial disruption of barrier integrity, permeability is spatially heterogeneous and often incomplete. Infiltrative margins frequently retain a relatively intact BBB, limiting immune cell trafficking and systemic drug penetration precisely where residual tumor cells persist after resection. Moreover, BBB disruption does not necessarily translate into productive immune engagement. Regions with increased permeability often coincide with hypoxia, aberrant angiogenesis, and dense infiltration of immunosuppressive macrophages [166]. This creates a paradox of “access without activation,” where therapeutic antibodies may enter but fail to overcome entrenched immune suppression. Beyond physical restriction, the BBB also shapes immune privilege within the central nervous system by regulating cytokine gradients, endothelial adhesion molecules, and leukocyte transmigration. Consequently, effective immunotherapy in GBM must address both the structural and functional dimensions of BBB-mediated immune regulation [167, 168] (Table 1).

**Table 1:** Translational strategies to overcome spatio-temporal immune resistance in GBM

Challenge	Biological Basis	Current Approaches	Emerging Strategies
Blood–brain barrier restriction [166]	Tight endothelial junctions, heterogeneous permeability [169]	High-dose systemic therapy [170]	Focused ultrasound BBB opening [171]
Spatial immune suppression [172]	Myeloid-dominant niches, TAM signaling [173]	Immune checkpoint inhibitors [174]	Myeloid reprogramming (CSF1R, PI3Kγ inhibition) [175]
Antigen heterogeneity [176]	Variable antigen expression across regions [52]	Single-antigen CAR-T or vaccines [176]	Multi-target CAR constructs [177, 178]
Clonal evolution [157]	Therapy-driven subclone selection [161]	Continuous therapy schedules [179]	Adaptive therapy and evolutionary modeling [62, 180]
Immune exhaustion [92, 181]	Chronic antigen stimulation [182]	PD-1/PD-L1 blockade [183, 184]	Combination checkpoint targeting (TIM-3, LAG-3) [185]
Poor drug penetration [186]	BBB and tumor architecture [187]	Systemic chemotherapy [188]	Convection-enhanced delivery (CED) [189]

#### Toward adaptive and spatially guided immunotherapy in glioblastoma

Given the limited penetration of systemic therapies across the blood–brain barrier (BBB), regionally targeted delivery strategies such as convection-enhanced delivery (CED) [189] and focused ultrasound (FUS) have emerged as promising approaches to enhance drug distribution in glioblastoma (GBM). CED enables direct intraparenchymal infusion under controlled pressure gradients, allowing broader intratumoral distribution of antibodies, oncolytic viruses, and immunomodulatory agents, whereas FUS combined [190] with circulating microbubbles provides a non-invasive method for transient BBB opening to facilitate targeted delivery of checkpoint inhibitors, cytokines, or nanoparticle therapeutics [191, 192]. However, both technologies face significant translational challenges, including technical complexity, variability in drug distribution, and the need for specialized infrastructure. Beyond spatial delivery, emerging treatment paradigms emphasize evolution-informed adaptive therapy, in which treatment intensity and combinations are dynamically adjusted to limit clonal selection and immune escape [157]. Implementation of such strategies will require longitudinal biomarker monitoring—including ctDNA dynamics, T-cell repertoire changes, spatial immune heterogeneity, and immunosuppressive signaling activation—to guide therapeutic modification. Integrating these data with computational approaches, including artificial intelligence and predictive modeling, may enable proactive identification of resistant niches and optimization of individualized treatment schedules. Importantly, combination immunotherapy strategies must be balanced with careful safety considerations, as immune activation within the central nervous system can amplify neuroinflammation and cerebral edema. Consequently, adaptive neuro-immuno-oncology frameworks that integrate spatial drug delivery, biomarker-guided therapy adjustment, and predictive computational modeling may provide a rational path toward more effective and personalized GBM management. Future therapeutic strategies for glioblastoma (GBM) are increasingly shifting from conventional static treatment paradigms toward an adaptive ecosystem–based framework that recognizes tumors as dynamic and evolving biological systems. Advances in spatial multi-omics, liquid biopsy technologies, and artificial intelligence–driven modeling are expected to enable continuous monitoring of tumor–immune interactions and clonal evolution across both spatial and temporal dimensions. Within this framework, computational approaches such as digital twin models and evolutionary simulations may allow prediction of immune escape and therapy resistance, facilitating adaptive treatment scheduling and personalized intervention strategies. Importantly, these concepts may extend beyond GBM to other central nervous system tumors, although differences in baseline immunogenicity, blood–brain barrier integrity, and immune composition will require tumor-specific adaptation. Ultimately, durable therapeutic benefit will likely depend on achieving sustained immune reprogramming through integrated targeting of tumor-intrinsic pathways, myeloid-mediated immunosuppression, metabolic constraints, and T-cell exhaustion. By integrating spatial precision, temporal adaptability, and biomarker-guided modulation, this adaptive neuro-immuno-oncology paradigm may provide a conceptual framework for improving long-term disease control in GBM.

## Future perspectives

### integrating spatial and evolutionary heterogeneity: the Spatio-temporal immune ecosystem model

**Fig. 3:**
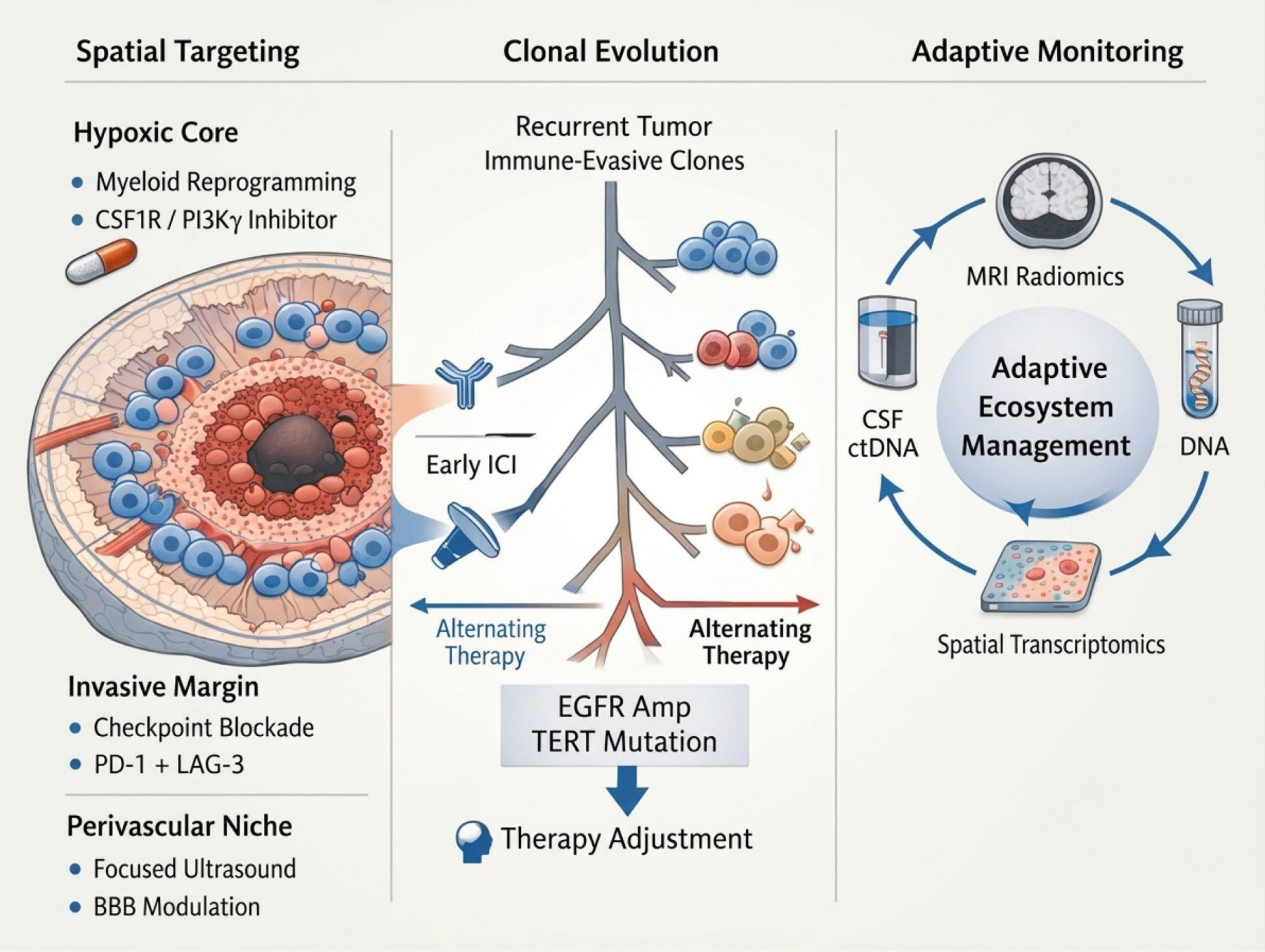
Adaptive, biomarker-guided immunotherapy within the spatio-temporal immune ecosystem of glioblastoma. Glioblastoma (GBM) is depicted as a dynamic spatio-temporal immune ecosystem in which spatial compartmentalization and clonal evolution jointly sustain therapeutic resistance. Anatomically distinct regions-including hypoxic cores, invasive margins, and perivascular niches-harbor heterogeneous immune states characterized by myeloid predominance, T-cell exhaustion, variable blood–brain barrier permeability, and metabolic stress, thereby creating region-specific immune constraints. In parallel, branching clonal evolution driven by trunk alterations such as EGFR amplification and TERT promoter mutation generates genetically and antigenically diverse subpopulations that undergo immune editing and therapy-induced selection, progressively favoring immune-evasive lineages at recurrence. The integration of multimodal biomarkers-including advanced radiomic imaging, cerebrospinal fluid–derived circulating tumor DNA, circulating T-cell receptor profiling, and spatial transcriptomics-enables longitudinal surveillance of both spatial immune architecture and temporal clonal dynamics. By linking ecosystem-level monitoring to iterative therapeutic adjustment, this framework shifts GBM management from static cytotoxic intervention toward adaptive ecosystem control aimed at disrupting immune inertia and delaying clonal escape

The Spatio-Temporal Immune Ecosystem Model reframes glioblastoma not as a uniformly immune-cold tumor but as a dynamically structured immune landscape shaped by the continuous interaction between spatial compartmentalization and temporal evolutionary adaptation (Fig. 3). Spatially, distinct tumor regions, including hypoxic cores, perivascular niches, and invasive margins, harbor heterogeneous immune states that impose selective pressures specific to each region [193]. Temporally, clonal diversification and immune editing reshape antigenicity and signaling pathways, progressively altering tumor–immune interactions [194]. The concept of “immune inertia” captures the self-reinforcing nature of resistance within this system. Spatial immune exclusion protects emerging resistant clones, while evolutionarily adapted clones further remodel their local microenvironments toward deeper immunosuppression, creating a positive feedback loop. As a result, resistance becomes stabilized across both axes [161, 195]. Breaking this cycle requires dual-axis intervention. Spatially precise delivery strategies can disrupt localized immune barriers, whereas evolution-informed adaptive scheduling can intercept clonal escape trajectories. By integrating these dimensions, the model provides a rational translational framework for designing next-generation biomarker-guided clinical trials in glioblastoma [154].

### The interdependence of spatial and temporal axes

Spatial heterogeneity and evolutionary heterogeneity in glioblastoma have traditionally been studied as distinct dimensions of tumor biology. However, emerging evidence indicates that these axes are deeply interdependent rather than independent phenomena. Spatial organization defines the ecological context in which clonal evolution unfolds [107]. Distinct tumor regions-hypoxic cores, perivascular niches, and infiltrative margins-impose region-specific selective pressures shaped by metabolic stress, vascular permeability, immune infiltration, and cytokine gradients. These microenvironmental forces influence which subclones survive, expand, or regress within each niche [196]. Conversely, evolutionary dynamics reshape spatial architecture over time. As immune-evasive or therapy-resistant subclones expand, they modify local cytokine networks, metabolic states, and immune cell recruitment patterns. In this manner, temporal adaptation reinforces spatial compartmentalization. Space seeds heterogeneity; time stabilizes and amplifies it. This reciprocal relationship transforms GBM into a dynamic, multi-dimensional system rather than a collection of independent tumor regions (Table 2) [204].

**Table 2:** Spatio-temporal immune ecosystem model in glioblastoma

Dimension	Molecular Level	Immune Consequence	Therapeutic Implication
Spatial	Regional gene expression gradients (hypoxia, angiogenesis, cytokine milieu)	Local immune suppression and functional compartmentalization [197]	Spatially targeted immunotherapy (intratumoral injection, convection-enhanced delivery, focused ultrasound) [111]
Evolutionary	Clonal diversification, neoantigen loss, interferon-pathway inactivation [198]	Progressive immune escape and temporal immunoediting [199]	Sequential/adaptive immunotherapy guided by longitudinal monitoring [200]
Integrated (Spatio-temporal)	Niche-specific evolution of tumor–immune interactions [201]	Self-reinforcing immune resistance landscape (“immune inertia”) [202]	Adaptive, real-time therapy optimization based on spatial transcriptomics + phylogenetic tracking [107, 203]

### Conceptualizing GBM as a Spatio-Temporal Immune Ecosystem

We propose the “spatio-temporal immune ecosystem” model to describe GBM as an evolving field of immune resistance structured by both anatomical compartmentalization and clonal diversification.

In this model, each spatial niche functions as a localized immune–tumor micro-ecosystem characterized by:

- Distinct gene expression gradients (hypoxia, angiogenesis, interferon signaling)
- Unique immune cell composition (myeloid dominance vs. T-cell infiltration)
- Differential vascular accessibility and metabolic stress
- Subclone-specific genomic and epigenetic states

Over time, these niches undergo iterative cycles of immune pressure and clonal adaptation. Immunogenic clones may be eliminated in interferon-rich margins, while immune-silent clones expand within hypoxic cores. Therapy-induced stress further perturbs this equilibrium, generating new selective bottlenecks. The cumulative result is a self-reinforcing landscape of immune inertia-a state in which immune suppression and evolutionary adaptation mutually stabilize each other. Rather than a uniformly “cold” tumor, GBM becomes a patchwork of temporally aged immune niches: some newly inflamed, others chronically suppressed, collectively sustaining resistance. This framework explains why uniform systemic therapies often fail: they confront a moving mosaic rather than a static target.

### Molecular and systems-level integration

At the molecular level, spatial–temporal integration can be conceptualized across three interconnected layers [154]:

- Genomic Layer: Branching clonal evolution generates subpopulations with distinct driver mutations, antigenicity, and therapy sensitivities.
- Microenvironmental Layer: Local immune composition and vascular topology shape the intensity and direction of selective pressures.
- Immunodynamic Layer: T-cell activation, exhaustion, and repertoire contraction evolve in parallel with tumor adaptation.

Systems biology approaches enable integration of these layers into predictive models. Spatial transcriptomics provides region-specific immune maps; multi-region sequencing reconstructs phylogenetic trees; longitudinal ctDNA and TCR sequencing track temporal shifts. Computational frameworks-including graph-based modeling and evolutionary game theory-can simulate how immune pressure propagates across spatial domains and predict emergent resistance fronts [205, 206]. Such integrative modeling reframes therapeutic planning from reactive intervention to anticipatory ecosystem management.

### Therapeutic implications of the spatio-temporal framework

Viewing GBM as a spatio-temporal immune ecosystem has profound therapeutic implications. First, spatial precision becomes essential. Immune-cold niches may require localized interventions-such as convection-enhanced delivery, focused ultrasound–mediated blood–brain barrier modulation [207], or perivascular targeting-to disrupt entrenched suppressive microenvironments [208]. Second, temporal adaptation is necessary. Continuous immune checkpoint blockade may select resistant clones; instead, adaptive scheduling that alternates selective pressures could delay fixation of immune-evasive populations. Evolution-informed sequencing of therapies may preserve immunogenic subclones while suppressing dominant resistant branches [209, 210]. Third, combination strategies must address both tumor-intrinsic drivers (e.g., RTK/PI3K signaling, DNA damage response pathways) and microenvironmental suppressors (e.g., myeloid polarization, metabolic competition). Multi-target interventions reduce the likelihood of escape through single-pathway adaptation [211] [212]. Finally, biomarker-guided monitoring-integrating imaging, tissue profiling, and liquid biopsy-can provide real-time feedback on ecosystem dynamics, enabling iterative adjustment of therapy. In this paradigm, immunotherapy is not a static addition to standard care but a dynamically modulated component within a broader ecosystem control strategy.

### Toward adaptive ecosystem management in GBM

**Fig. 4:**
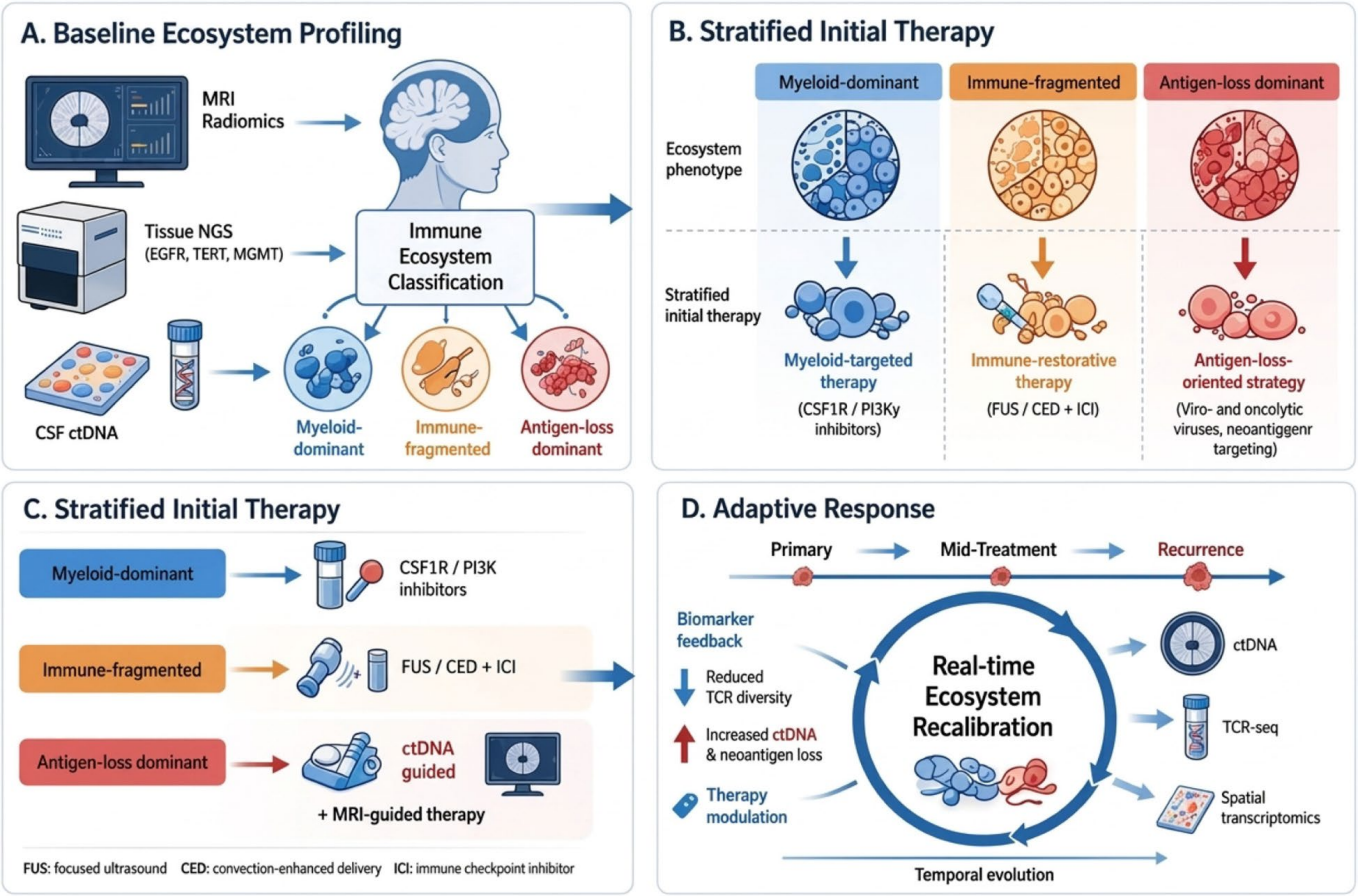
Ecosystem-guided clinical trial architecture for adaptive immunotherapy in glioblastoma. A biomarker-integrated clinical framework is proposed to operationalize the spatio-temporal immune ecosystem model in glioblastoma (GBM). At baseline, multimodal profiling—including MRI radiomics, tissue-based next-generation sequencing (EGFR, TERT, MGMT), cerebrospinal fluid circulating tumor DNA (ctDNA), and immune landscape classification—defines a tumor ecosystem phenotype characterized by spatial immune architecture and clonal composition. Based on this stratification, patients are assigned phenotype-directed initial therapy targeting dominant biological constraints, such as myeloid-mediated suppression, immune fragmentation, or antigen-loss–associated immune escape. During treatment, longitudinal biomarker feedback—including ctDNA dynamics, T-cell receptor diversity, radiomic evolution, and spatial transcriptomic signals—enables real-time ecosystem recalibration. Iterative therapy modulation across primary treatment, mid-course assessment, and recurrence aims to prevent fixation of resistant subclones and maintain immune-responsive tumor states. This adaptive trial architecture reframes GBM management from fixed protocol-driven therapy to dynamic ecosystem control guided by continuous molecular and immunologic surveillance

The spatio-temporal immune ecosystem model shifts the conceptual goal of GBM therapy from eradication of a homogeneous mass to management of a complex adaptive system. Complete elimination of all tumor cells may be unrealistic; instead, durable control may depend on preventing dominance of highly resistant subclones and maintaining immune-sensitive states. By integrating spatial mapping, evolutionary monitoring, and computational prediction, future treatment paradigms may resemble adaptive ecological management-where interventions are timed and localized based on evolving system states [213]. Such a framework does not negate the challenges of GBM but provides a structured lens through which therapeutic resistance can be understood and strategically countered [214]. The convergence of spatial compartmentalization and evolutionary adaptation explains the persistent failure of uniform therapeutic approaches in glioblastoma. Recognizing GBM as a spatio-temporal immune ecosystem offers a coherent conceptual scaffold linking molecular pathogenesis, immune dynamics, and clinical resistance. In the final section, we outline future directions and translational priorities for implementing adaptive, ecosystem-informed neuroimmuno-oncology (Fig. 4).

## Data Availability

No new data were generated or analyzed in this study. Data sharing is not applicable to this article.
